# Radiation dosimetry of ^18^F-AzaFol: A first in-human use of a folate receptor PET tracer

**DOI:** 10.1186/s13550-020-00624-2

**Published:** 2020-04-08

**Authors:** Silvano Gnesin, Joachim Müller, Irene A. Burger, Alexander Meisel, Marco Siano, Martin Früh, Matthias Choschzick, Cristina Müller, Roger Schibli, Simon M. Ametamey, Philipp A. Kaufmann, Valerie Treyer, John O. Prior, Niklaus Schaefer

**Affiliations:** 1 Institute of Radiation Physics, Lausanne University Hospital and University of Lausanne, Lausanne, Switzerland; 2 Department of Radiology and Nuclear Medicine, Cantonal Hospital St. Gallen, St. Gallen, Switzerland; 3 Department of Nuclear Medicine, University Hospital of Zurich, Zurich, Switzerland; 4 Department of Nuclear Medicine, Kantonsspital Baden, Baden, Switzerland; 5 Department of Chemistry and Applied Biosciences, ETH Zurich, Zurich, Switzerland; 6 Department of Internal Medicine—Hematology & Oncology, Stadtspital Waid, Zurich, Switzerland; 7 Department of Oncology and Hematology, Cantonal Hospital St. Gallen, St. Gallen, Switzerland; 8 University of Bern, Bern, Switzerland; 9 Institute for Pathology and Molecular Pathology, University Hospital of Zurich, Zurich, Switzerland; 10 Center for Radiopharmaceutical Sciences ETH-PSI-USZ, Paul Scherrer Institute, Villigen-PSI, Villigen, Switzerland; 11 Department of Nuclear Medicine and Molecular Imaging, Lausanne University Hospital and University of Lausanne, Rue du Bugnon 46, CH-1011 Lausanne, Switzerland

**Keywords:** Folate receptor, FOLR1, FRalpha, FRα, ^18^F-Azafol, Imaging, Positron emission tomography (PET), OLINDA/EXM, Dosimetry, Lung cancer, Choroid plexuses

## Abstract

**Background:**

The folate receptor alpha (FRα) is an interesting target for imaging and therapy of different cancers. We present the first in-human radiation dosimetry and radiation safety results acquired within a prospective, multicentric trial (NCT03242993) evaluating the ^18^F-AzaFol (3′-aza-2′-[^18^F]fluorofolic acid) as the first clinically assessed PET tracer targeting the FRα.

**Material and methods:**

Six eligible patients presented a histologically confirmed adenocarcinoma of the lung with measurable lesions (≥ 10 mm according to RECIST 1.1). TOF-PET images were acquired at 3, 11, 18, 30, 40, 50, and 60 min after the intravenous injection of 327 MBq (range 299–399 MBq) of ^18^F-AzaFol to establish dosimetry. Organ absorbed doses (AD), tumor AD, and patient effective doses (E) were assessed using the OLINDA/EXM v.2.0 software and compared with pre-clinical results.

**Results:**

No serious related adverse events were observed. The highest AD were in the liver, the kidneys, the urinary bladder, and the spleen (51.9, 45.8, 39.1, and 35.4 μGy/MBq, respectively). Estimated patient and gender-averaged E were 18.0 ± 2.6 and 19.7 ± 1.4 μSv/MBq, respectively. E in-human exceeded the value of 14.0 μSv/MBq extrapolated from pre-clinical data. Average tumor AD was 34.8 μGy/MBq (range 13.6–60.5 μGy/MBq).

**Conclusions:**

^18^F-Azafol is a PET agent with favorable dosimetric properties and a reasonable radiation dose burden for patients which merits further evaluation to assess its performance.

**Trial registration:**

ClinicalTrial.gov, NCT03242993, posted on August 8, 2017

## Background

The folate receptor-alpha (FRα) is a transmembrane receptor overexpressed in multiple tumor types and has been explored as a novel target for cancer treatments [1–3]. However, recent trials evaluating FRα-directed antibodies or drug conjugates, such as farletuzumab and vintafolide, did not show any benefit in ovarian cancer patients in phase III studies [4, 5]. The reasons for these negative results were certainly multiple but may be partially explained by heterogenous antigen expression in the cancer tissue and possible off-target binding.

To better predict the FR-related accumulation of a possible FRα-ligand, molecular imaging is a valuable option, which has the advantage to provide a spatial distribution of the disease burden. Pre-treatment imaging is able to evaluate the target distribution regarding homogeneity and if any unforeseen binding (sink effect) might influence the uptake of the targeted agent [6]. A number of FRα-targeting imaging agents have been evaluated to address this issue. Etarfolatide, a ^99m^Tc complexed folic acid conjugate has been tested clinically to enable selection of patients prior to Vintafolide treatment [7, 8]. However, Etarfolatide is a single-photon emission tomography (SPECT) tracer with limited imaging resolution and inferior quantification compared to positron emission tomography (PET). In pre-clinical studies, folate-derived ^68^Ga-based PET probe has shown slightly superior in vivo performance in comparison to Etarfolide [9]. This compound is to the best of our knowledge under investigation in humans in an inflammatory indication (NCT03494114) and currently not published in a peer-reviewed journal. At the Center for Radiopharmaceutical Sciences (Villigen-PSI, Switzerland), a series of ^18^F-based folate tracers have been developed over the past decade [10], which resulted recently in a so-called “integrated” approach where the ^18^F-label was directly introduced in the folic acid backbone [11]. Promising pre-clinical results led to a further optimization and development of an ^18^F-based folic acid tracer, 3′-aza-2′-[^18^F]fluorofolic acid (herein referred to as ^18^F-AzaFol), which can be synthesized in an easy-to-perform one-step labeling approach suitable for an automated synthesis module [12].

Here, we present the first publication of a human dosimetry study of ^18^F-AzaFol and overall the first clinical results of a PET radiotracer for FR-imaging in oncological patients.

## Materials and methods

### Study design

The present study was designed to establish the ^18^F-AzaFol PET/CT dosimetry as a radiation safety cohort in patients with adenocarcinoma of the lung. It is part of a larger, prospective, multi-centric study conducted in three Swiss hospitals. The study was approved by the respective institutions’ ethics committee, the national health authorities, centrally monitored by a dedicated, independent Contract Research Organization and listed in the trial list of the National Institute of Health Trial database (NCT03242993).

### Patients

Eligible patients had a histologically confirmed non-small cell lung carcinoma of the lung with measurable lesions (≥ 10 mm according to RECIST 1.1 [13]) and were staged by standard of care imaging with an indication for systemic treatment. The last systemic treatment should not have been applied within 3 weeks before performing the study exam. Male and female patients needed to be 18 years or older as well as voluntarily sign the informed consent form. Exclusion criteria were, in brief, any contraindications to the class of drugs under study, pregnancy or breast-feeding, clinically significant concomitant disease states (e.g., renal failure, hepatic dysfunction, or cardiovascular disease) and poor performance status (ECOG > 2). Patients were asked to discontinue taking vitamin supplements containing folic acid 48 h prior to the PET/CT. Due to pre-clinical results established by our group, all patients received a single bolus injection of 1 mg of folic acid before tracer administration [12].

### Radiochemistry

The precursor (protected 3′-aza-2′-chloro-folate) was synthesized by an external manufacturer (Merck & Cie, Schaffhausen, Switzerland). Radiolabeling and subsequent global deprotection of the protected 3′-aza-2′-chloro-folate precursor resulted in 3′-aza-2′-[^18^F]fluorofolic acid herein referred to as ^18^F-AzaFol. Radiolabeling of the folate precursor was achieved within 17 min at 150 °C in dimethyl sulfoxide (≥ 99.7%, over molecular sieve, Acros Organics) and the protective groups were subsequently cleaved under acidic conditions (4 M hydrochloric acid, Merck EMPROVE®) at 60 °C. The product was purified over two solid-phase extraction cartridges (30 mg mixed-mode cation exchange sorbent for bases, particle size 30 μm (MCX), Oasis) and subsequently eluted with 50 mM phosphate buffer containing 10% methanol (pH 7.4). The desired compound was then isolated by isocratic HPLC (eluent, 20 mM phosphate buffer containing 7% methanol pH 7.4; column, Luna 5 μm PFP(2) 100 Å, 250 × 10 mm, Phenomenex; flow rate, 4 mL/min.; retention time, 11 to 12 min). After further purification over a third MCX cartridge, formulation was achieved by eluting the product from the MCX cartridge with 5 mL of 50 mM phosphate buffer containing 10% ethanol (pH 7.4) into the bulk vial, which was prefilled with 9 mL of saline. The bulk product was then aseptically dispensed through a sterile filter (Millex-GV, 0.22 μm, PVDF, diameter 33 mm, Millipore) to yield the final, sterile, and non-pyrogenic product with a total activity of approximately 2 GBq in 6 mL plus samples for quality control, including sterility testing and a retained sample. Radiochemical purity was ≥ 95% and molar activity ≥ 100 GBq/μmol. The analytical HPLC was run with an isocratic method (column, XSelect HSS PFP XP, 100 Å, 2.5 μm, 4.6 mm × 150 mm (Waters); eluent, 10 mM ammonium acetate buffer, pH 6.2 containing 5% methanol; flow rate, 1 mL/min; UV, 280 nm; radio detector, 2 × 2″ NaI (Tl) crystal). The retention time of ^18^F-AzaFol was 7.8 min.

### PET/CT acquisition protocol

Seven PET images (from the top of the skull to the mid femora, 1 min/bed position, six bed positions, total scan length was 6 min) were acquired on a Discovery MI time-of-flight (TOF) PET/CT (GE Healthcare, Waukesha, WI, USA) at the University Hospital Zurich. All pertinent corrections (normalization, dead-time, physical decay correction at the start of the PET scan, random coincidence, attenuation, and scatter correction) were applied. PET field-of-view width was 70 cm with 256 × 256 image discretization. The voxel size in the axial direction and transverse plane were, respectively, 2.79 mm and 2.73 mm. Each patient underwent a low-dose (120 kVp, 15 mA/s, spiral pitch = 0.98, 40 mm beam collimation, CTDI_vol_ = 1.3 mGy) whole-body CT for attenuation correction prior to the first PET acquisition.

PET data were reconstructed using a proprietary three-dimensional ordered subset expectation-maximization algorithm (3 iterations × 16 subsets) with TOF information and PSF recovery correction (VPFXs, vendor-based reconstruction algorithm) with 6.4-FWHM Gaussian post-reconstruction filtering.

Patients were instructed to void the urinary bladder after the 1 h scan period.

### Organ segmentation

Co-registered PET and CT data were loaded using PMOD 3.9 (PMOD Technologies, Zurich, Switzerland). Volumes of interest (VOI) were manually drawn slice-by-slice on the axial plane of the CT part of each PET/CT study using the polygonal segmentation tool of PMOD by two operators in consensus (SG, NS) for the following body regions: brain, thyroid, lungs, heart, liver, spleen, stomach, kidneys, prostate (in men), red marrow, intestines, and whole-body. Tumors with increased folate receptor expression were manually segmented on CT data using the combined information of PET/CT and prior standard of care clinical imaging.

The urinary bladder was manually segmented on the emission PET data to account for possible changes of volume due to bladder filling between PET/CT acquisitions.

Specific biological uptake was found in the choroid plexuses. This small vascular structure was segmented by emission-based threshold segmentation to avoid important PET signal spill-out. We adopted a threshold of 5% of the maximum signal intensity to delineate the choroids VOI. This relatively low threshold level was possible considering the negligible tracer uptake of the surrounding cerebral tissue. An example of source organ VOI segmentation is provided in Supplemental Data 1 (Figure S1).

### Absorbed dose (AD) estimations

The total activity contained in each considered source organ was obtained by multiplying the average activity concentration (expressed in Bq/mL) by the organ volume expressed in milliliters and normalized to the administered patient activity at each time point. For all source organs, normalized time-activity curves (nTAC) were obtained assuming an initial constant uptake (*A*_organ_ (*t*) = *A*_organ_ (*t* = 3 min) for 0 min ≤ *t* < 3 min) and thus using a bi-exponential fit of experimental data extended from *t* = 3 min and the last measured data point (60 min post-administration). Exponential fit parameters were obtained using the kinetic module of OLINDA/EXM v.2.1. Beyond, in absence of late measured data, a simple mono-exponential physical decay was assumed to derive time-integrated activity coefficients (TIACs) by analytical time integration of source organ time-activity curves using MATLAB software (release 2017a; The MathWorks, Inc., Natick, MA, USA).

Bone marrow dosimetry was estimated by combining information from different VOIs as previously reported [14]. In brief, the red marrow time-integrated activity coefficient (TIAC) was obtained from nTAC in which the red marrow activity concentration (Bq/mL) was sampled in VOIs drawn in the humeral bone, the heads of femora, and lumbar vertebrae (L3–L4). The total activity in the red bone marrow was obtained by multiplying the average activity concentration measured in the sampled VOIs by the total red marrow mass of ICRP-89 adult male/female reference phantoms [15], which was repeated for each patient and at each acquired time point.

To estimate the colon AD, the total number of colonic disintegrations was partitioned to its components (right colon, left colon, and rectum) proportionally to their respective masses of the ICRP-89 male and female reference phantoms [15].

The total amount of radioactivity excreted from the body through the urine was not quantitatively assessed as no urine samples were collected in this study. We estimated the total amount of radioactivity present in the urinary bladder at 1 h after administration (latest quantitative PET acquisition available). This amount of radioactivity was assumed to be completely voided just after the last PET acquisition, when the patient was allowed voiding at the toilet. To obtain the TIAC for the urinary bladder, the excreted fraction (the voided activity at 1 h divided by the patient-administered activity) was used in input to the special kinetic module of OLINDA/EXM 2.1 for the urinary bladder voiding using a voiding period of 1 h. We adopted this methodology in the absence of urine samples and PET acquisition for *t* > 60 min.

The TIAC for the rest of the body was obtained by subtracting the sum of the source organ TIACs from the whole-body TIAC.

TIACs were used in input to the OLINDA/EXM® 2.0 code (HERMES Medical Solution AB, Stockholm, Sweden) [15] that provided organ AD and effective dose (E) per unit of administered activity in μGy/MBq and μSv/MBq, respectively, using the NURBS voxel-based phantoms [16] adjusted to the ICRP-89 organ masses [17] and ICRP103 tissue weighting factors (w_T_) [18]. According to publication ICRP 103, E is calculated as the weighted average of organ/tissue equivalent doses, summing equivalent doses multiplied by tissue weighting factors (w_T_), which provide a simplified representation of fractional contributions to total stochastic detriment from cancer and heritable effects. While exposures may relate to individuals or population groups, E is calculated for reference persons. For a general population, ICRP recommends to average E computed for a reference male and a reference female phantom. We will call this quantity “gender-averaged E.” In this paper, we will also compute E for specific patients, which we will refer as “patient E” using the reference organ masses of OLINDA/EXM 2.0, thus adopting a methodological approach typical of radiation protection, where the dosimetry of a reference adult subject is the focus.

A specific dose assessment was performed for the choroid plexuses and tumors, which do not appear in the list of available source/target organs in OLINDA. Trapezoidal time integration of the normalized time-activity curve, for 0 ≤ *t* ≤ 60 min, was performed using MATLAB software (release 2017a; The MathWorks, Inc., Natick, MA, USA), analytical integration assuming mono-exponential physical decay was applied beyond. To account for loss of signal due to partial volume effects (PVE), tumor TIACs were corrected using recovery coefficients (RC) obtained in a NEMA/IEC NU2 phantom experiment using the same clinical acquisition and reconstruction parameters used for the patient study as reported in Supplemental material (Supplemental Data 2). TIACs for the choroid and tumors were used as input in the OLINDA sphere model. For the choroids, we also applied a more realistic AD estimation using the Monte Carlo-derived analytical approach proposed by Amato et al. [19] in which the complex geometry of this organ was approximated using a set of simple geometrical structures such as (cylinders and parallelepipeds). The mass of the choroids was estimated to be 2.76 g and 1.81 g for male and female as previously reported in [20].

We performed an additional analysis to investigate the influence of the nTAC extrapolation to infinity on source organ TIACs. At this scope, in addition to the assumption of pure physical decay from *t* > 60 min, we also computed mean source organ TIACs (obtained from the time integration of averaged nTACs across the six patients) by assuming mono- and bi-exponential prolongation to infinity of the nTACs.

Coefficients of determination (*R*^2^) were computed to evaluate the goodness of both mono-exponential and bi-exponential nTAC fits for source organs exhibiting a monotonic decreasing uptake or decreasing activity accumulation with time (all considered source organs except the urinary bladder).

### Tumor contrast

We characterized the tumor to background contrast as a function of the time elapsed after radiotracer administration; with this aim, we computed for each acquired PET the tumor to lung ratio (T/L) by dividing the average activity concentration measured in lung tumor VOIs by the average activity concentration measured in the lung VOI. We report the average ± SD of T/L obtained for nine tumors.

## Results

### Patients

Six patients with non-small cell lung cancer were included in this dosimetric study (2 women, 4 men; average age 72 ± 10 years, average body mass 73 ± 15 kg and average height 1.67 ± 0.07 m). Participant demography and injection data are reported in Supplemental Data 3. Each patient underwent 7 consecutive whole-body TOF PET/CT acquisitions at 3 ± 1, 11 ± 1, 18 ± 1, 30 ± 3, 40 ± 3, 50 ± 1, and 60 ± 3 min after the intravenous administration of 327 ± 37 MBq (range 299–399 MBq) of ^18^F-AzaFol. All injections were well tolerated. No immediate symptoms or modification of vital signs were observed.

### Imaging

**Fig. 1:**
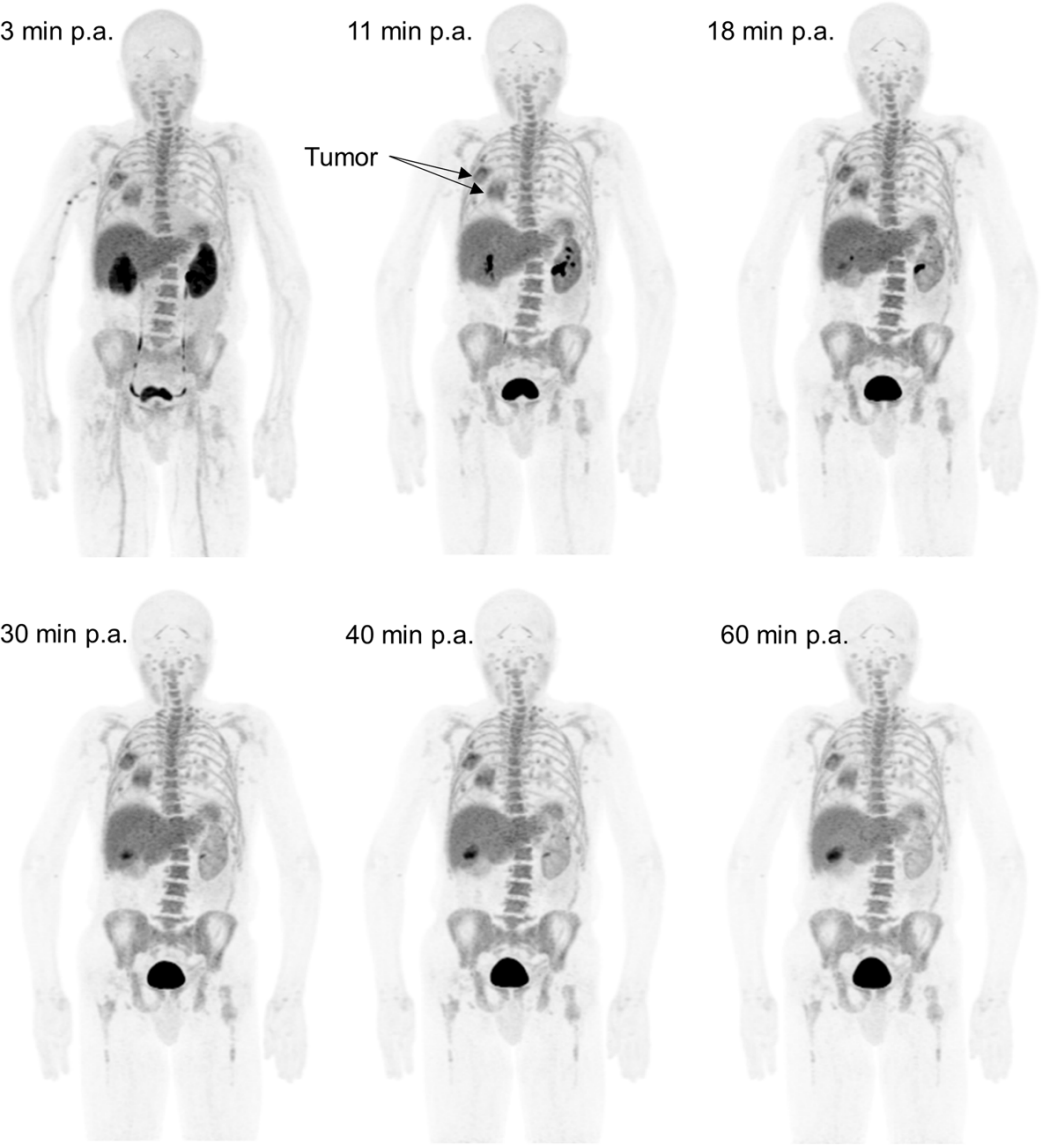
Example of maximum intensity projections 3, 11, 18, 30, 40, and 60 min post-tracer administration (p.a) showing in-patient ^18^F-Azafol activity distribution

**Fig. 2:**
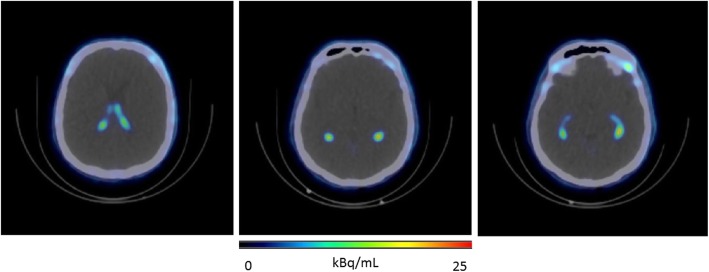
Typical ^18^F-AzaFol uptake pattern in the choroid plexuses (lateral ventricles) for a representative patient at 30 min post-administration

A typical biodistribution of ^18^F-AzaFol is shown in Fig. 1 (MIP images at 60 min for the six patents are shown in Figure S4 of Supplemental Data 4). The main way of biological excretion of the radiopharmaceutical from the body was through the urine. Prominent kidney uptake was visible in the early phase followed by rapid renal wash-out. Significant urinary bladder activity accumulation was seen as early as 10 min after injection. A significant uptake was observed in the choroid plexuses from the early time point images (Fig. 2).

### Absorbed dose estimations of ^18^F-AzaFol in humans

**Fig. 3:**
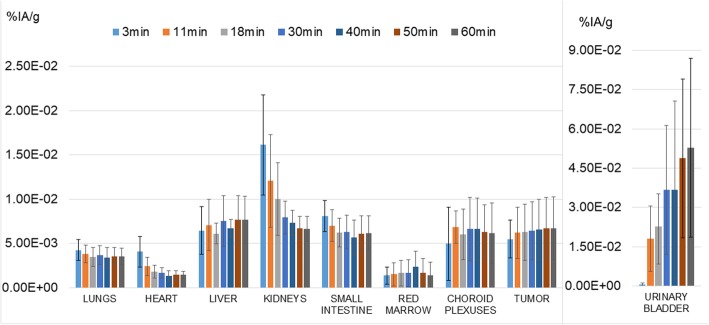
Biological organ kinetic of ^18^F-AzaFol corrected for ^18^F physical decay. Color bars represent the average percent of injected activity per gram of tissue (%IA/g) ± 1SD, for each time point

**Fig. 4:**
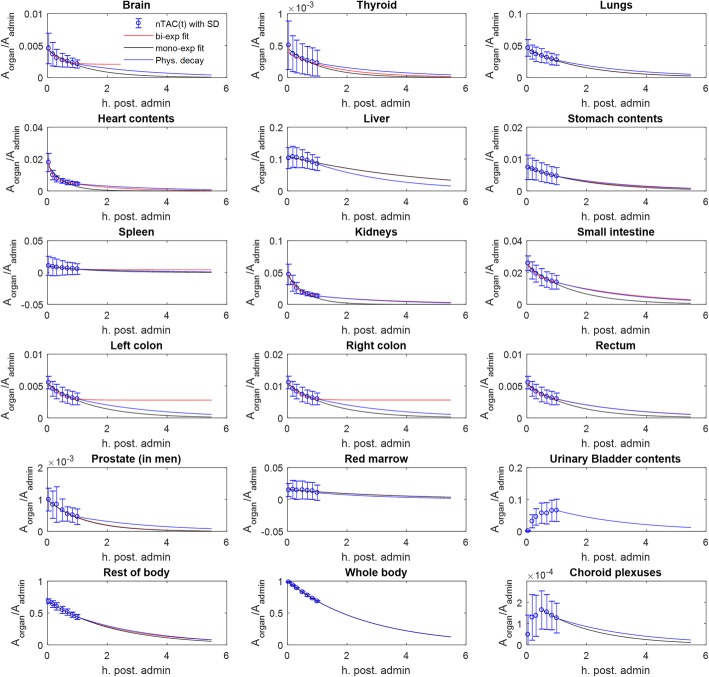
Mean nA ± SD (average values and SD evaluated across the 6 patients), for considered source organs. Mono- and bi-exponential fits of experimental data are displayed in addition to the tail according to pure physical decay to infinity for *t* > 60 min. For sake of visibility, the time base in each source organ graph was restricted to three times the physical half-life of ^18^F

Bio-kinetic data (percent of injected activity normalized by the source organ mass at different measured time points), for relevant source organs, the choroid plexuses and the tumors, corrected for ^18^F physical decay are shown in Fig. 3. nTAC data for the six patients considered in this study were reported as supplemental material (Supplemental Data 5). Figure 4 shows, for each considered source organ, mean normalize activity (nA) ± SD (average values and SD evaluated across all six patients), mono-, and bi-exponential fits, in addition to the tail representing pure physical decay to infinity for *t* > 60 min. Table 1 reported the *R*^2^ values of mono- and bi-exponential nTAC fits. In most source organs, higher R^2^ values were obtained adopting a bi-exponential fit of experimental data.

**Table 1:** Goodness of fit expressed by the coefficient of determination *R*^2^ for considered source organs average nA values fitted with mono- and bi-exponential functions (averaged nA and fits are visible in Fig. 1). The average nA were computed from experimental data collected across the six-patient dataset

Source organ	*R*^2^ bi-exp	*R*^2^ mono-exp
Brain	0.994	0.932
Thyroid	0.962	0.881
Lungs	0.962	0.962
Heart contents	1.000	0.861
Liver	0.856	0.856
Stomach contents	1.000	1.000
Spleen	0.997	0.982
Kidneys	1.000	0.949
Small intestine	0.999	0.946
Left colon	0.996	0.945
Right colon	0.996	0.943
Rectum	0.999	0.945
Prostate (in men)	0.968	0.961
Red marrow	0.662	0.662
Rest of the body	0.999	0.999
Whole body	1.000	1.000

Computed source organ TIACs are reported in Table 2. Organ AD, patient E, as well as the extrapolated E for the reference person, are reported in Table 3. The organ receiving the highest AD (mean ± SD) was the liver (51.9 ± 16.4 μGy/MBq), followed by the kidneys, the urinary bladder wall, and the spleen (45.8 ± 8.3, 39.1 ± 16.8 and 35.4 ± 39.7 μGy/MBq respectively). Using a 1-h urinary voiding cycle, we obtained an averaged E of 18.0 ± 2.6 μSv/MBq in our patient cohort. The corresponding E for the reference person was 19.7 ± 1.4 μSv/MBq. AD in tumor was 34.8 ± 17 μGy/MBq (range 13.6-60.5 μGy/MBq). The estimated AD in the choroid plexuses, for the two dosimetry approaches used (OLINDA spherical model and Monte Carlo-derived analytical calculation), are reported in Table 4.

**Table 2:** Time-integrated activity coefficients (TIAC in units of MBq h/MBq) for source organs considered in the dosimetry study. Two male patients had surgical prostate resection before inclusion in this study

Source organ/patient (gender)	1 (F)	2 (M)	3 (F)	4 (M)	5 (M)	6 (M)	Average	SD
Brain	1.30E− 02	6.60E− 03	1.15E− 02	7.20E− 03	6.30E− 03	6.60E− 03	8.53E− 03	2.93E− 03
Thyroid	3.00E− 04	8.00E− 04	4.00E− 04	8.00E− 04	2.50E− 03	5.00E− 04	8.83E− 04	8.18E− 04
Lungs	1.11E− 01	1.09E− 01	7.88E− 02	1.58E− 01	1.13E− 01	7.04E− 02	1.07E− 01	3.08E− 02
Heart	2.02E− 02	2.31E− 02	2.33E− 02	1.60E− 02	1.45E− 02	1.89E− 02	1.93E− 02	3.61E− 03
Liver	4.73E− 01	2.72E− 01	2.63E− 01	2.98E− 01	2.58E− 01	3.70E− 01	3.22E− 01	8.48E− 02
Stomach	3.54E− 02	7.70E− 03	1.82E− 02	2.19E− 02	8.50E− 03	1.78E− 02	1.83E− 02	1.01E− 02
Spleen	1.09E− 02	6.60E− 03	8.60E− 03	6.50E− 03	7.80E− 03	9.09E− 02	2.19E− 02	3.38E− 02
Kidneys	5.02E− 02	5.10E− 02	7.68E− 02	4.97E− 02	6.96E− 02	5.37E− 02	5.85E− 02	1.17E− 02
Small Intestine	6.66E− 02	4.96E− 02	3.00E− 02	5.38E− 02	4.94E− 02	7.89E− 02	5.47E− 02	1.67E− 02
Left colon	1.42E− 02	1.06E− 02	6.50E− 03	1.15E− 02	1.06E− 02	1.69E− 02	1.17E− 02	3.54E− 03
Right colon	2.85E− 02	2.12E− 02	1.29E− 02	2.31E− 02	2.12E− 02	3.38E− 02	2.35E− 02	7.13E− 03
Rectum	1.42E− 02	1.06E− 02	6.50E− 03	1.15E− 02	1.06E− 02	1.69E− 02	1.17E− 02	3.54E− 03
Prostate (in men)	–	2.20E− 03	–	–	1.10E− 03	–	1.65E− 03	7.78E− 04
Red marrow	1.31E− 02	2.42E− 02	3.38E− 02	2.03E− 02	1.33E− 01	2.86E− 02	4.22E− 02	4.50E− 02
Urinary bladder	8.12E− 02	5.49E− 02	8.82E− 02	4.76E− 02	5.08E− 02	5.74E− 03	5.47E− 02	2.93E− 02
Rest of the body	1.46E+ 00	1.82E+ 00	1.72E+ 00	1.77E+ 00	1.71E+ 00	1.81E+ 00	1.71E+ 00	1.33E− 01

*M* male, *F* female

**Table 3:** Average organ AD and E according to OLINDA/EXM 2.0 for a 1-h urinary voiding cycle. Reference organ masses for the adult male (M) and adult female (F) Reference phantoms of OLINDA/EXM 2.0 were used

Organ AD in mGy/MBq								
Organs/patient	1 (F)	2 (M)	3 (F)	4 (M)	5 (M)	6 (M)	Average	SD
Adrenals	2.61E− 02	1.93E− 02	2.27E− 02	2.00E− 02	2.06E− 02	2.52E− 02	2.23E− 02	2.84E− 03
Brain	4.67E− 03	3.26E− 03	4.80E− 03	3.32E− 03	3.36E− 03	3.27E− 03	3.78E− 03	7.42E− 04
Breasts	9.68E− 03	–	1.01E− 02	–	–	–	9.89E− 03	2.97E− 04
Esophagus	1.63E− 02	1.29E− 02	1.45E− 02	1.37E− 02	1.25E− 02	1.37E− 02	1.39E− 02	1.35E− 03
Eyes	7.77E− 03	7.76E− 03	9.04E− 03	7.58E− 03	7.57E− 03	7.73E− 03	7.91E− 03	5.61E− 04
Gallbladder wall	2.33E− 02	2.05E− 02	1.97E− 02	2.15E− 02	2.00E− 02	2.47E− 02	2.16E− 02	1.99E− 03
Left colon	2.89E− 02	2.45E− 02	2.15E− 02	2.56E− 02	2.46E− 02	3.32E− 02	2.64E− 02	4.10E− 03
Small intestine	3.44E− 02	2.54E− 02	2.32E− 02	2.64E− 02	2.53E− 02	3.37E− 02	2.81E− 02	4.76E− 03
Stomach wall	3.05E− 02	1.57E− 02	2.23E− 02	2.19E− 02	1.57E− 02	2.22E− 02	2.14E− 02	5.47E− 03
Right colon	3.09E− 02	2.62E− 02	2.19E− 02	2.74E− 02	2.60E− 02	3.50E− 02	2.79E− 02	4.52E− 03
Rectum	3.05E− 02	2.42E− 02	2.39E− 02	2.48E− 02	2.41E− 02	3.06E− 02	2.64E− 02	3.27E− 03
Heart wall	2.04E− 02	1.78E− 02	2.06E− 02	1.72E− 02	1.57E− 02	1.78E− 02	1.83E− 02	1.91E− 03
Kidneys	4.54E− 02	3.85E− 02	6.01E− 02	3.80E− 02	4.96E− 02	4.31E− 02	4.58E− 02	8.25E− 03
Liver	8.34E− 02	4.08E− 02	4.95E− 02	4.45E− 02	3.92E− 02	5.38E− 02	5.19E− 02	1.64E− 02
Lungs	2.84E− 02	2.19E− 02	2.19E− 02	2.89E− 02	2.25E− 02	1.77E− 02	2.36E− 02	4.31E− 03
Ovaries	1.46E− 02	–	1.56E− 02	–	–	–	1.51E− 02	7.07E− 04
Pancreas	2.30E− 02	1.55E− 02	1.94E− 02	1.65E− 02	1.53E− 02	1.87E− 02	1.81E− 02	2.94E− 03
Prostate	–	2.98E− 02	–	1.30E− 02	1.93E− 02	1.95E− 02	2.04E− 02	6.96E− 03
Salivary glands	8.62E− 03	9.16E− 03	9.91E− 03	8.99E− 03	8.88E− 03	9.13E− 03	9.12E− 03	4.36E− 04
Red marrow	1.26E− 02	1.17E− 02	1.47E− 02	1.15E− 02	1.89E− 02	1.23E− 02	1.36E− 02	2.83E− 03
Osteogenic cells	9.65E− 03	9.61E− 03	1.11E− 02	9.44E− 03	1.36E− 02	1.01E− 02	1.06E− 02	1.60E− 03
Spleen	2.56E− 02	1.55E− 02	2.22E− 02	1.58E− 02	1.73E− 02	1.16E− 01	3.54E− 02	3.97E− 02
Testes	–	9.50E− 03	–	9.20E− 03	9.04E− 03	9.14E− 03	9.22E− 03	1.98E− 04
Thymus	1.35E− 02	1.16E− 02	1.36E− 02	1.19E− 02	1.11E− 02	1.14E− 02	1.22E− 02	1.09E− 03
Thyroid	9.26E− 03	1.28E− 02	1.02E− 02	1.32E− 02	2.56E− 02	1.03E− 02	1.36E− 02	6.10E− 03
Urinary bladder wall	5.54E− 02	3.72E− 02	6.00E− 02	3.34E− 02	3.48E− 02	1.36E− 02	3.91E− 02	1.68E− 02
Uterus	1.77E− 02	–	1.83E− 02	–	–	–	1.80E− 02	4.24E− 04
Total body	1.39E− 02	1.11E− 02	1.37E− 02	1.11E− 02	1.11E− 02	1.16E− 02	1.21E− 02	1.35E− 03
**Patient E (w**_**T**_**ICRP-103) mSv/MBq**	**2.28E− 02**	**1.53E− 02**	**1.91E− 02**	**1.70E− 02**	**1.67E− 02**	**1.73E− 02**	**1.80E− 02**	**2.62E− 03**
**Reference person E** **(w**_**T**_**ICRP-103) mSv/MBq**	**2.16E− 02**	**1.82E− 02**	**1.80E− 02**	**2.01E− 02**	**1.98E− 02**	**2.05E− 02**	**1.97E− 02**	**1.38E− 03**

**Table 4:** Dosimetry assessment in choroid plexuses. The average TIAC value was used in input to the OLINDA/EXM sphere model and to the Monte Carlo-derived analytical model [17]

Choroid plexuses TIAC (MBq h/MBq)			
Average	Min	Max	SD
4.62E− 04	3.72E− 04	5.15E− 04	5.81E− 05
**Choroid plexuses AD (μGy/MBq)**			
**OLINDA sphere model, M (2.76 g)**	**OLINDA sphere model, F (1.81 g)**	**M-C Analytic, M (2.76 g)**	**M-C Analytic, F (1.81 g)**
2.47E− 02	3.69E− 02	2.25E− 02	3.22E− 02

### Tumor uptake and contrast

We analyzed nine lung tumors across the six patient population. The average tumor uptake in terms of percentage-injected activity per gram (%IA/g) corrected for ^18^F physical decay is indicated in Fig. 3. Averaged %IA/g monotonically increased over time, nearly reaching a plateau within the 60 min after radiopharmaceutical administration. Individual tumor TACs and %IA/g are shown in supplemental material Figure S6 (Supplemental Data 6).

We measured the T/L on nine lung tumors. As shown in Table 5, the average T/L varied between 3.5 at the time of the first PET acquisition and remains between 4.8 and 5 for PET acquisition time ≥ 40 min where the T/L reach a plateau. Based on these results, we estimate an optimal acquisition PET time between 40 min and 1 h post-administration.

**Table 5:** T/L measured for nine lung tumors as a function of the time elapsed after radiopharmaceutical administration. In bracket, we indicated the patient identifier

Time (min post-admin)	3	11	18	30	40	50	60
T1 (P1)	2.22	2.32	2.42	2.37	2.49	2.35	2.39
T2 (P3)	2.58	3.04	3.43	3.03	3.71	3.65	3.39
T3 (P3)	2.67	3.88	4.44	4.14	4.86	5.06	4.65
T4 (P4)	4.60	5.72	5.89	6.19	6.75	6.62	6.59
T5 (P4)	4.90	6.04	6.23	6.65	7.21	7.08	7.08
T6 (P5)	1.74	2.42	2.67	2.93	2.75	2.77	2.71
T7 (P5)	5.79	8.41	9.27	9.21	8.90	8.90	8.87
T8 (P5)	1.78	2.00	2.09	2.06	2.14	2.07	2.00
T9 (P6)	5.32	5.06	4.89	5.22	5.17	5.89	5.65
**Average**	**3.51**	**4.32**	**4.59**	**4.65**	**4.89**	**4.93**	**4.81**
**SD**	**1.62**	**2.15**	**2.30**	**2.38**	**2.35**	**2.38**	**2.39**

## Discussion

To the best of our knowledge, this is the first clinical study publication describing a FR-targeting PET agent.

Our data show the liver to be the most irradiated organ in ^18^F-AzaFol PET imaging, followed by the kidneys, the urinary bladder wall, and the spleen, with a mean E of 18.0 ± 2.6 μSv/MBq corresponding to an effective dose of 5.9 ± 0.8 mSv (range 5.4–7.2 mSv). The reference person E was 19.7 ± 1.4 μSv/MBq, which is comparable to the radiation burden of 19.0 μSv/MBq for ^18^F-FDG PET imaging [21, 22].

Measured PET data were obtained during the first hour after radiopharmaceutical administration and the source organ TIACs were obtained by performing analytical time integration of nTAC bi-exponential fits for 3 min ≤ *t* ≤ 60 min. In this time interval, in fact, the bi-exponential fits were characterized by a (slightly) higher *R*^2^ compared to the mono-exponential fits. In the absence of information for 0 ≤ *t* < 3 min, we decided to assume a constant organ uptake with a pure mono-exponential physical decay (with the characteristic ^18^F half-life of 110 min) for *t* > 60 min. This approach has been adopted when lacking later PET acquisitions and potentially leads to conservative (over-) estimates of the actual organ AD and E. As reported in Fig. 4, for some source organs, the bi-exponential fits exhibited a late effective half-life higher than the ^18^F physical half-life, thus resulting in unrealistic TIAC (and AD) extrapolations when the time integration was extended to infinity. Another possible option would be consisted in adopting a mono-exponential extension to infinity of source organ nTACs. This approach would result in an average of 20% lower TIACs, but, because of the lack of experimental data for *t* > 60 min, we preferred to keep a conservative approach by adopting the physical decay to infinity.

Tumors and choroidal plexuses showed an initially growing uptake followed by a late decrease; this fact motivated our adoption of a trapezoidal numerical integration for *t* ≤ 60 min.

We derived RCs from a NEMA/IEC phantom experiment (provided in Supplemental Data 2) and used them to correct tumor TIACs to compensate for PVE. Among considered source organs, only the thyroid had a size (male thyroid ICRP-89 volume = 20, mL, RC (20 mL) = 0.77) that would motivate the use of PVE compensation. This compensation was not applied. Nevertheless, the thyroid exhibited very little (and unspecific) radiotracer uptake with consequent minor impact on E determination (3% of E).

In a phase-I study using ^99m^Tc-Etarfolatide, (22) Yamada et al [23], reported an E of 8.9 μSv/MBq, translating into an E of 6.6 mSv for a standard administration of 740 MBq. This E is therefore comparable to the E we reported for our PET tracer.

We computed doses according to OLINDA/EXM 2.0 reference-phantom organ masses. This approach is applied in the low-dose range typical of radiopharmaceuticals used for diagnostic purposes in which the patient’s radiation protection is the main concern, considering the fact that E is a metric used for assessing stochastic risks in a population.

It is known that folic acid plays a prominent role in the development, function, and repair of the central nervous system [24]. Choroid plexuses, a vascular structure localized in the brain ventricles and responsible for producing the cerebrospinal fluid, is involved in maintaining the folate concentration gradient in that fluid. Specific folate uptake by choroidal plexuses epithelial cells was already reported in rat’s models [25, 26] and folate receptors in tumors of the choroid plexus were proposed as potential targets for radiation therapy in mice [27]. ^18^F-AzaFol showed a specific biological uptake in choroid plexuses. Notably, choroid plexuses appear to still be in the biological uptake phase at the end of the acquisition time (Fig. 3). Estimated TIAC for the choroid plexuses with ^18^F-AzaFol (4.62E− 04 MBq h/MBq) is higher than TIAC previously reported in [20] where a ^68^Ga-RGD compound was used (3.08E− 04 MBq h/MBq). Nevertheless, when considering the same organ mass and geometry (with OLINDA sphere model and a mass of 2.76 g) used for the AD calculation, the AD estimate to the choroid plexuses for the ^18^F-AzaFol (2.47E− 02 μGy/MBq) was lower than the dose estimated of the ^68^Ga-RGD compound (3.96E− 02 μGy/MBq). This fact can be explained by the lower average energy of the β^+^-emission of ^18^F compared to ^68^Ga (0.25 vs. 0.83 MeV). Although there are no specific dose constraints for the choroid plexuses in external beam radiotherapy for instance, in our opinion, the relatively high doses delivered to these structures warrants further consideration, especially in case of any therapeutic applications of folate-based radioconjugates.

In our dose extrapolation from previously published pre-clinical data [12], the kidneys followed by the liver were the organs with the highest exposure. This is the opposite of what was obtained in the direct human dosimetry. Although, as reported elsewhere [28], the order of the most irradiated organs can differ between human-measured dosimetry and its extrapolation from murine bio-kinetic data.

The E extrapolated from pre-clinical data was predicted to be 14.0 μSv/MBq, which did underestimate the measured human E of 19.7 μSv/MBq by 29%. Such large discrepancies are not uncommon [29, 30]. The underestimation could be explained by a generally slower metabolism and elimination of the radiation carrier substance in humans compared to mice [31]. In addition, available mice bio-kinetic data missed of dosimetric information for some source organ, such as the urinary bladder, which also contributed significantly to the overall E. Also, note that the mice data used to extrapolate the human absorbed dose were collected at only three time points that differed from the human data points (30, 60 and 90 min vs. 3, 11, 18, 30, 40, 50 and 60 after injection respectively). These methodological differences could partly contribute to the aforementioned absorbed dose differences, a reality which highlights the importance of performing dedicated in-human dosimetry studies to complement pre-clinical results prior to any clinical use of radiopharmaceuticals.

Recent developments in the field of FRα-based therapies such as chimeric antigen receptor T (CAR-T) cell therapy in acute myeloid leukemia [32], triple-negative breast cancer [33], gastric cancer [34], or ovarian cancer [35] are very promising. Pre-clinical and clinical results suggest that besides careful clinical patient selection, such therapies might benefit from the application of the best possible FRα-based imaging agent to improve further patient selection [10]. ^18^F-AzaFol is a ready-to-use diagnostic radiopharmaceutical, which could be integrated into many ongoing clinical programs with antibody-drug conjugates (e.g., mirvetuximab-soravtansine) or CAR-T cells. Overall, we believe our novel PET tracer ^18^F-AzaFol to be very promising regarding a clinical application, where it might serve as a companion diagnostic tool for FRα-targeting anticancer drugs. Furthermore, our data were obtained in a multicentric, prospective study underlining the feasibility of larger trials.

However, it has to be critically acknowledged that this dosimetric study has several limitations. First, it has a small number of patients, which is however comparable to other dosimetric studies and it is part of a larger prospective trial being currently analyzed. Secondly, we could not acquire data after 60 min because of total dose constraints imposed by the regulating authorities. As discussed above, this most likely leads to overestimated doses due to our conservative approach of extrapolating the physical decay to infinity. Furthermore, our untreated patient population was symptomatic by pain and shortness of breath and imaging > 60 min was very challenging and ethically questionable. Third, our trial was open for lung adenocarcinoma and ovarian cancer. This is due to a known overexpression of these tumors in previously published histology studies [36]. There are also reported overexpression in other malignancies which were not addressed in our trial due to a most possible sound trial design and homogeneity of the patient population. In this initial dosimetry study, we only included lung cancer patients despite this trial was open for lung cancer and ovarian cancer. This is because of the need of urgent chemotherapy initiation in patients with recurrent ovarian cancer.

## Conclusions

In conclusion, our study showed that ^18^F-AzaFol is a safe PET agent with good dosimetric properties with a reasonable radiation dose burden for patients. Considering the large number of ongoing clinical trials evaluating FRα-targeted cancer drugs, this imaging tracer merits further investigation of its clinical potential as a predictive biomarker.

## Supplementary information

**Additional file 1: Figure S1.** Segmented VOI obtained with PMOD software co-registered on the CT of the patient represented in Figure 1.

**Additional file 2: S2. Supplementary Material**

**Additional file 3: S3.** Participants and injections data.

**Additional file 4: Figure S4.** Patient MIP images at 60 min using the same SUV scale for all images (0–5 g/mL).

**Additional file 5: S5. Supplementary Material**

**Additional file 6: Figure S6.** Time-activity curves (TAC), %IA/g and %IA/g corrected for ^18^F physical decay for all tumor analyzed (T#). In bracket, we indicated the patient identifier (P#).

## Data Availability

The datasets used and/or analyzed during the current study are available from the corresponding author on reasonable request.

## Abbreviations

PET: Positron emission tomography
FRα: Folate Receptor-alpha
TOF: Time-of-flight
CT: Computed tomography
AD: Absorbed dose
E: Effective dose
SPECT: Single-photon emission tomography
SD: Standard deviation
nTAC: Normalized time-activity curve
nA: Normalize activity
FDG: Fluorodeoxyglucose
TIAC: Time-integrated activity coefficient
RC: Recovery coefficient
NEMA: National Electrical Manufacturers Association
IEC: International Electrotechnical Commission
ICRP: International Commission on Radiobiological Protection
PVE: Partial volume effect
RGD: Arginine-glycine-aspartic acid
CAR: Chimeric antigen receptor
HPLC: High-performance liquid chromatography
MCX: Mixed-mode cationic exchange
FWHM: Full width at half maximum
CTDI: Computed tomography dose index
VOI: Volume of interest
NURBS: Non-uniform rational B-spline
w_T_: Weighting factor
