# Supplementary material for: Radiation dosimetry of 18F-AzaFol: A first in-human use of a folate receptor PET tracer
Source: EJNMMI Res. 2020 Apr 8;10:32. doi: 10.1186/s13550-020-00624-2 (PMC7142191; doi:10.1186/s13550-020-00624-2)

**Figure S1.** Segmented VOI obtained with PMOD software co-registered on the CT of the patient represented in Figure 1.


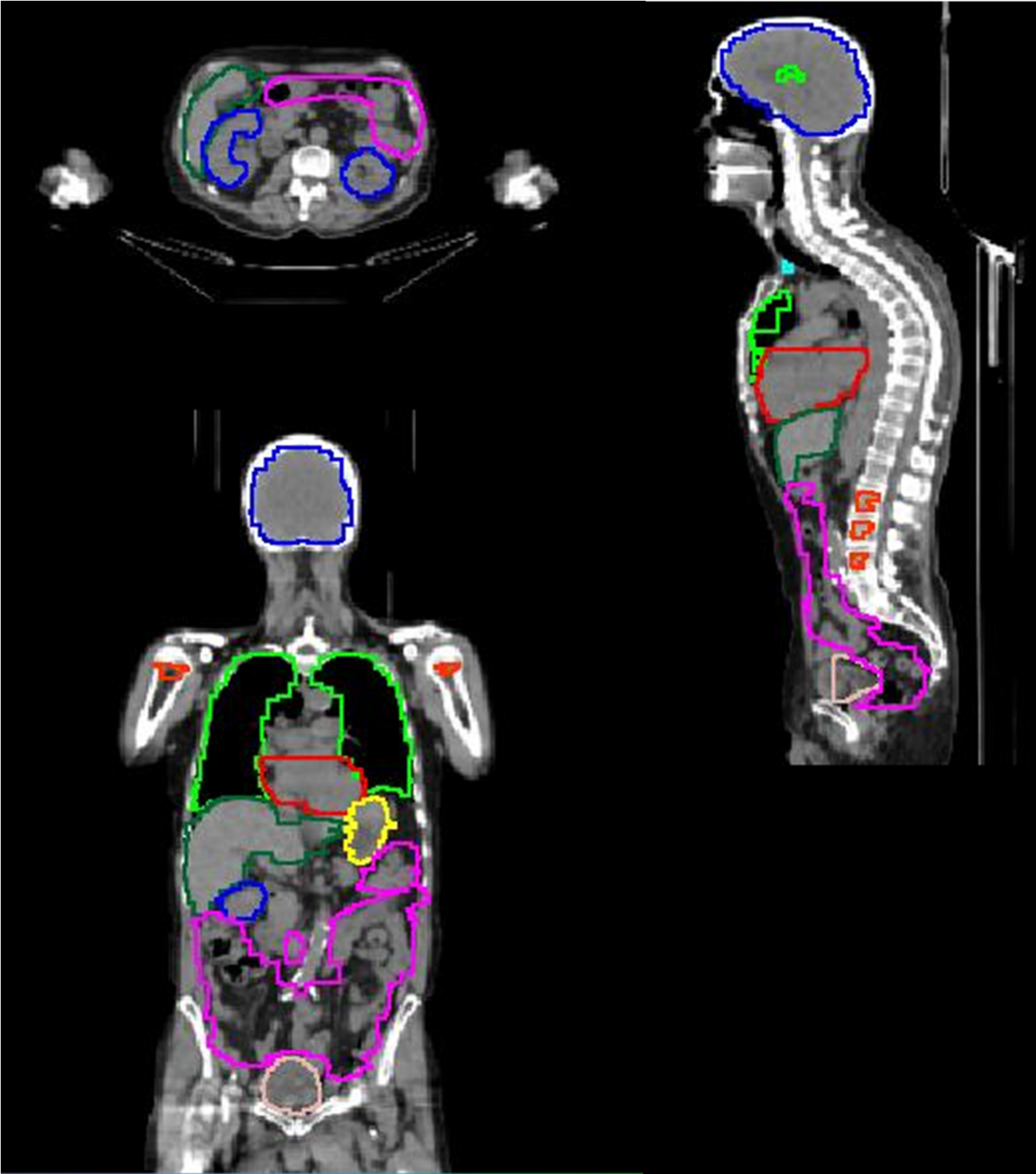

Supplement: Supplementary file 1 — Additional file 1: Figure S1. Segmented VOI obtained with PMOD software co-registered on the CT of the patient represented in Figure 1. [file 13550_2020_624_MOESM1_ESM.docx]
