# Supplementary material for: Radiation dosimetry of 18F-AzaFol: A first in-human use of a folate receptor PET tracer
Source: EJNMMI Res. 2020 Apr 8;10:32. doi: 10.1186/s13550-020-00624-2 (PMC7142191; doi:10.1186/s13550-020-00624-2)
Supplement: Supplementary file 2 — Additional file 2: S2. Supplementary Material [file 13550_2020_624_MOESM2_ESM.docx]

**S2. Supplementary Material**

We obtained recovery coefficients (RC) as a function of size from a phantom experiment in which a NEMA/IEC NU2 test object was filled with 5kBq/mL in the main volume (9.7L) while spherical inserts were filled with a 5× higher activity concentration. The size and RC values obtained for the six spherical inserts are reported in the table here below. The acquired PET phantom data was reconstructed using the same parameters used for patients.

| **sphere size ( Ø in mm)** | **10** | **13** | **17** | **22** | **28** | **37** |
| --- | --- | --- | --- | --- | --- | --- |
| sphere volume (mL) | 0.52 | 1.15 | 2.57 | 5.57 | 11.49 | 26.51 |
| RC | 0.43 | 0.57 | 0.66 | 0.73 | 0.77 | 0.76 |

RC data were fitted with a hyperbole function: RC(sph_vol) = C1–C2/(sph_vol +C3) as displayed in the figure here below. The fit coefficients (C1= 0.7854, C2= –0.3572 and C3=0.4863) were obtained by minimizing a cost function implemented in the fminsearch function of Matlab used at this scope. The coefficient of determination of the fit was R^2^=0.995.
