# Supplementary material for: Radiation dosimetry of 18F-AzaFol: A first in-human use of a folate receptor PET tracer
Source: EJNMMI Res. 2020 Apr 8;10:32. doi: 10.1186/s13550-020-00624-2 (PMC7142191; doi:10.1186/s13550-020-00624-2)
Supplement: Supplementary file 3 — Additional file 3: S3. Participants and injections data. [file 13550_2020_624_MOESM3_ESM.docx]

**S3.** Participants and injections data.

| **Patient ID** | **Height (cm)** | **Weight (kg)** | **Age**  **(y)** | **Activity (MBq)** | **Injected Mass (µg)** | **Normalized injected mass (µg/kg=** |
| --- | --- | --- | --- | --- | --- | --- |
| P1 | 162 | 59 | 72 | 326 | 10.6 | 0.180 |
| P2 | 178 | 75 | 75 | 322 | 6.7 | 0.089 |
| P3 | 158 | 64 | 78 | 299 | 4.8 | 0.075 |
| P4 | 187 | 85 | 52 | 318 | 6.2 | 0.073 |
| P5 | 158 | 60 | 73 | 299 | 6.1 | 0.102 |
| P6 | 168 | 118 | 70 | 332 | 5.7 | 0.048 |
