# Supplementary figures and images for: Radiation dosimetry of 18F-AzaFol: A first in-human use of a folate receptor PET tracer
Source: EJNMMI Res. 2020 Apr 8;10:32. doi: 10.1186/s13550-020-00624-2 (PMC7142191; doi:10.1186/s13550-020-00624-2)

**Figure S4.** Patient MIP images at 60 min using the same SUV scale for all images (0–5 g/mL).


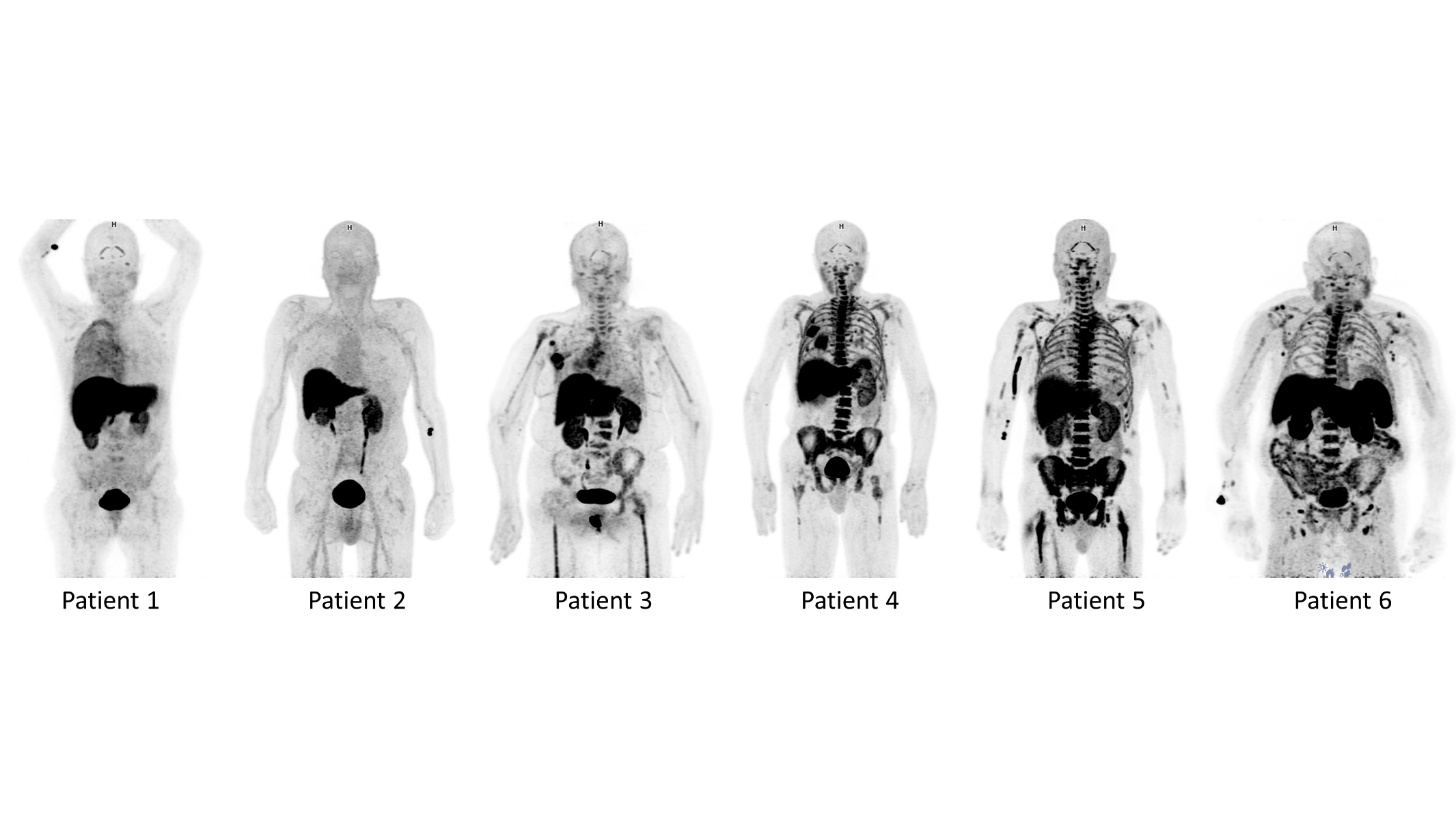

Supplement: Supplementary file 4 — Additional file 4: Figure S4. Patient MIP images at 60 min using the same SUV scale for all images (0–5 g/mL). [file 13550_2020_624_MOESM4_ESM.docx]
