# Supplementary material for: Radiation dosimetry of 18F-AzaFol: A first in-human use of a folate receptor PET tracer
Source: EJNMMI Res. 2020 Apr 8;10:32. doi: 10.1186/s13550-020-00624-2 (PMC7142191; doi:10.1186/s13550-020-00624-2)
Supplement: Supplementary file 5 — Additional file 5: S5. Supplementary Material [file 13550_2020_624_MOESM5_ESM.docx]

**S5. Supplementary Material**

| **Source organ / h after admin** | **0.04** | **0.21** | **0.37** | **0.53** | **0.69** | **0.85** | **1.02** |
| --- | --- | --- | --- | --- | --- | --- | --- |
| Brain | 8.22E-03 | 6.09E-03 | 5.11E-03 | 4.35E-03 | 3.85E-03 | 3.44E-03 | 3.04E-03 |
| Thyroid | 2.32E-04 | 1.57E-04 | 1.35E-04 | 1.19E-04 | 1.18E-04 | 9.45E-05 | 8.58E-05 |
| Lungs | 4.87E-02 | 4.17E-02 | 3.81E-02 | 3.52E-02 | 3.24E-02 | 3.01E-02 | 2.78E-02 |
| Heart contents | 1.75E-02 | 9.97E-03 | 7.70E-03 | 6.50E-03 | 5.50E-03 | 5.01E-03 | 4.60E-03 |
| Liver | 1.56E-01 | 1.62E-01 | 1.56E-01 | 1.49E-01 | 1.41E-01 | 1.31E-01 | 1.23E-01 |
| Stomach contents | 1.40E-02 | 1.22E-02 | 1.13E-02 | 1.06E-02 | 1.00E-02 | 9.35E-03 | 9.16E-03 |
| Spleen | 6.75E-03 | 4.60E-03 | 3.84E-03 | 3.42E-03 | 3.07E-03 | 2.82E-03 | 2.66E-03 |
| Kidneys | 4.83E-02 | 2.93E-02 | 2.04E-02 | 1.64E-02 | 1.45E-02 | 1.24E-02 | 1.08E-02 |
| Small intestine | 3.09E-02 | 2.44E-02 | 2.13E-02 | 1.96E-02 | 1.86E-02 | 1.76E-02 | 1.70E-02 |
| Left colon | 6.62E-03 | 5.22E-03 | 4.57E-03 | 4.20E-03 | 3.98E-03 | 3.77E-03 | 3.64E-03 |
| Right colon | 1.32E-02 | 1.04E-02 | 9.14E-03 | 8.40E-03 | 7.97E-03 | 7.55E-03 | 7.28E-03 |
| Rectum | 6.62E-03 | 5.22E-03 | 4.57E-03 | 4.20E-03 | 3.98E-03 | 3.77E-03 | 3.64E-03 |
| Prostate (in men) | - | - | - | - | - | - | - |
| Red marrow | 2.78E-03 | 3.97E-03 | 4.14E-03 | 4.08E-03 | 3.89E-03 | 3.72E-03 | 3.56E-03 |
| Urinary Bladder contents | 1.97E-03 | 5.30E-02 | 7.36E-02 | 9.02E-02 | 9.16E-02 | 8.97E-02 | 9.75E-02 |
| Rest of body | 6.22E-01 | 5.57E-01 | 5.10E-01 | 4.62E-01 | 4.29E-01 | 4.03E-01 | 3.67E-01 |
| Whole body | 9.84E-01 | 9.25E-01 | 8.70E-01 | 8.18E-01 | 7.69E-01 | 7.23E-01 | 6.80E-01 |

**Patient 1:** Normalized activity in source organs for the seven acquired PET scans.

| **Source organ / h after admin** | **0.04** | **0.18** | **0.31** | **0.44** | **0.57** | **0.83** | **0.96** |
| --- | --- | --- | --- | --- | --- | --- | --- |
| Brain | 3.59E-03 | 2.90E-03 | 2.55E-03 | 2.32E-03 | 2.10E-03 | 1.77E-03 | 1.63E-03 |
| Thyroid | 4.99E-04 | 3.82E-04 | 3.19E-04 | 2.95E-04 | 2.56E-04 | 2.10E-04 | 1.99E-04 |
| Lungs | 5.92E-02 | 4.63E-02 | 4.05E-02 | 3.60E-02 | 3.31E-02 | 2.94E-02 | 2.76E-02 |
| Heart contents | 1.80E-02 | 1.13E-02 | 9.54E-03 | 8.30E-03 | 7.19E-03 | 5.95E-03 | 5.46E-03 |
| Liver | 7.00E-02 | 7.44E-02 | 7.86E-02 | 7.96E-02 | 8.01E-02 | 7.74E-02 | 7.60E-02 |
| Stomach contents | 4.21E-03 | 3.75E-03 | 3.32E-03 | 2.95E-03 | 2.45E-03 | 1.95E-03 | 1.85E-03 |
| Spleen | 4.13E-03 | 2.93E-03 | 2.54E-03 | 2.29E-03 | 2.12E-03 | 1.70E-03 | 1.63E-03 |
| Kidneys | 3.18E-02 | 2.21E-02 | 1.89E-02 | 1.63E-02 | 1.49E-02 | 1.31E-02 | 1.28E-02 |
| Small intestine | 2.54E-02 | 2.19E-02 | 1.94E-02 | 1.70E-02 | 1.55E-02 | 1.30E-02 | 1.24E-02 |
| Left colon | 5.45E-03 | 4.70E-03 | 4.15E-03 | 3.64E-03 | 3.32E-03 | 2.78E-03 | 2.66E-03 |
| Right colon | 1.09E-02 | 9.40E-03 | 8.29E-03 | 7.28E-03 | 6.64E-03 | 5.57E-03 | 5.33E-03 |
| Rectum | 5.45E-03 | 4.70E-03 | 4.15E-03 | 3.64E-03 | 3.32E-03 | 2.78E-03 | 2.66E-03 |
| Prostate (in men) | 1.16E-03 | 1.01E-03 | 9.27E-04 | 8.68E-04 | 8.00E-04 | 5.60E-04 | 5.32E-04 |
| Red marrow | 1.20E-02 | 9.17E-03 | 9.17E-03 | 7.76E-03 | 7.07E-03 | 6.56E-03 | 6.24E-03 |
| Urinary Bladder contents | 0.00E+00 | 1.81E-02 | 3.39E-02 | 4.52E-02 | 5.18E-02 | 6.57E-02 | 6.60E-02 |
| Rest of body | 7.31E-01 | 7.01E-01 | 6.53E-01 | 6.13E-01 | 5.75E-01 | 5.01E-01 | 4.71E-01 |
| Whole body | 9.84E-01 | 9.35E-01 | 8.90E-01 | 8.47E-01 | 8.06E-01 | 7.30E-01 | 6.95E-01 |

**Patient 2:** Normalized activity in source organs for the seven acquired PET scans.

| **Source organ / h after admin** | **0.06** | **0.18** | **0.29** | **0.52** | **0.64** | **0.86** | **0.97** |
| --- | --- | --- | --- | --- | --- | --- | --- |
| Brain | 6.75E-03 | 5.91E-03 | 4.37E-03 | 3.71E-03 | 3.48E-03 | 3.01E-03 | 2.79E-03 |
| Thyroid | 2.86E-04 | 1.90E-04 | 1.75E-04 | 1.30E-04 | 1.20E-04 | 1.08E-04 | 1.02E-04 |
| Lungs | 4.05E-02 | 3.16E-02 | 2.57E-02 | 2.60E-02 | 2.43E-02 | 1.99E-02 | 2.01E-02 |
| Heart contents | 2.42E-02 | 1.44E-02 | 9.68E-03 | 7.61E-03 | 6.83E-03 | 5.54E-03 | 5.14E-03 |
| Liver | 8.64E-02 | 8.92E-02 | 7.72E-02 | 8.24E-02 | 7.95E-02 | 7.25E-02 | 7.02E-02 |
| Stomach contents | 7.98E-03 | 7.70E-03 | 7.09E-03 | 5.61E-03 | 5.61E-03 | 4.98E-03 | 4.61E-03 |
| Spleen | 5.09E-03 | 3.92E-03 | 2.88E-03 | 2.69E-03 | 2.54E-03 | 2.24E-03 | 2.15E-03 |
| Kidneys | 5.55E-02 | 5.64E-02 | 4.20E-02 | 2.50E-02 | 2.16E-02 | 1.76E-02 | 1.69E-02 |
| Small intestine | 1.98E-02 | 1.47E-02 | 1.29E-02 | 9.32E-03 | 8.06E-03 | 7.14E-03 | 7.30E-03 |
| Left colon | 4.24E-03 | 3.16E-03 | 2.76E-03 | 2.00E-03 | 1.73E-03 | 1.53E-03 | 1.57E-03 |
| Right colon | 8.49E-03 | 6.32E-03 | 5.52E-03 | 4.00E-03 | 3.45E-03 | 3.06E-03 | 3.13E-03 |
| Rectum | 4.24E-03 | 3.16E-03 | 2.76E-03 | 2.00E-03 | 1.73E-03 | 1.53E-03 | 1.57E-03 |
| Prostate (in men) | - | - | - | - | - | - | - |
| Red marrow | 2.07E-02 | 1.83E-02 | 1.36E-02 | 1.95E-02 | 1.86E-02 | 1.77E-02 | 6.70E-03 |
| Urinary Bladder contents | 0.00E+00 | 2.74E-02 | 4.97E-02 | 7.81E-02 | 9.07E-02 | 1.02E-01 | 1.06E-01 |
| Rest of body | 6.90E-01 | 6.50E-01 | 6.36E-01 | 5.51E-01 | 5.17E-01 | 4.63E-01 | 4.44E-01 |
| Whole body | 9.76E-01 | 9.34E-01 | 8.95E-01 | 8.20E-01 | 7.86E-01 | 7.23E-01 | 6.93E-01 |

**Patient 3:** Normalized activity in source organs for the seven acquired PET scans.

| **Source organ / h after admin** | **0.04** | **0.18** | **0.31** | **0.44** | **0.71** | **0.84** | **0.97** |
| --- | --- | --- | --- | --- | --- | --- | --- |
| Brain | 3.78E-03 | 2.81E-03 | 2.47E-03 | 2.24E-03 | 1.94E-03 | 1.90E-03 | 1.84E-03 |
| Thyroid | 4.62E-04 | 3.38E-04 | 3.05E-04 | 2.82E-04 | 2.48E-04 | 2.29E-04 | 2.18E-04 |
| Lungs | 5.87E-02 | 5.52E-02 | 5.40E-02 | 5.28E-02 | 4.57E-02 | 4.36E-02 | 4.12E-02 |
| Heart contents | 2.33E-02 | 1.06E-02 | 6.80E-03 | 5.43E-03 | 3.99E-03 | 3.70E-03 | 3.33E-03 |
| Liver | 9.44E-02 | 9.80E-02 | 9.89E-02 | 9.43E-02 | 8.86E-02 | 8.50E-02 | 7.95E-02 |
| Stomach contents | 7.20E-03 | 7.16E-03 | 7.18E-03 | 6.97E-03 | 6.39E-03 | 6.03E-03 | 5.82E-03 |
| Spleen | 2.84E-03 | 2.36E-03 | 2.12E-03 | 2.02E-03 | 1.85E-03 | 1.77E-03 | 1.71E-03 |
| Kidneys | 4.28E-02 | 2.68E-02 | 2.12E-02 | 1.72E-02 | 1.35E-02 | 1.24E-02 | 1.15E-02 |
| Small intestine | 2.67E-02 | 2.08E-02 | 1.83E-02 | 1.69E-02 | 1.51E-02 | 1.45E-02 | 1.39E-02 |
| Left colon | 5.72E-03 | 4.45E-03 | 3.91E-03 | 3.63E-03 | 3.23E-03 | 3.10E-03 | 2.98E-03 |
| Right colon | 1.14E-02 | 8.91E-03 | 7.83E-03 | 7.26E-03 | 6.45E-03 | 6.19E-03 | 5.97E-03 |
| Rectum | 5.72E-03 | 4.45E-03 | 3.91E-03 | 3.63E-03 | 3.23E-03 | 3.10E-03 | 2.98E-03 |
| Prostate (in men) | - | - | - | - | - | - | - |
| Red marrow | 9.55E-03 | 7.86E-03 | 7.00E-03 | 6.46E-03 | 5.67E-03 | 5.40E-03 | 5.24E-03 |
| Urinary Bladder contents | 2.81E-03 | 4.01E-02 | 5.22E-02 | 5.51E-02 | 3.23E-02 | 5.79E-02 | 5.72E-02 |
| Rest of body | 6.90E-01 | 6.45E-01 | 6.03E-01 | 5.72E-01 | 5.36E-01 | 4.82E-01 | 4.59E-01 |
| Whole body | 9.85E-01 | 9.35E-01 | 8.89E-01 | 8.46E-01 | 7.64E-01 | 7.27E-01 | 6.92E-01 |

**Patient 4:** Normalized activity in source organs for the seven acquired PET scans.

| **Source organ / h after admin** | **0.03** | **0.16** | **0.29** | **0.54** | **0.67** | **0.80** | **1.05** |
| --- | --- | --- | --- | --- | --- | --- | --- |
| Brain | 2.87E-03 | 2.24E-03 | 2.05E-03 | 1.87E-03 | 1.79E-03 | 1.75E-03 | 1.61E-03 |
| Thyroid | 1.25E-03 | 9.17E-04 | 8.33E-04 | 7.48E-04 | 7.01E-04 | 6.78E-04 | 6.25E-04 |
| Lungs | 4.61E-02 | 4.00E-02 | 3.75E-02 | 3.42E-02 | 3.18E-02 | 3.15E-02 | 2.89E-02 |
| Heart contents | 1.56E-02 | 6.20E-03 | 4.70E-03 | 4.05E-03 | 3.51E-03 | 3.68E-03 | 3.47E-03 |
| Liver | 8.26E-02 | 8.75E-02 | 8.73E-02 | 8.06E-02 | 7.62E-02 | 7.28E-02 | 6.65E-02 |
| Stomach contents | 2.96E-03 | 2.79E-03 | 2.84E-03 | 2.59E-03 | 2.50E-03 | 2.42E-03 | 2.17E-03 |
| Spleen | 3.31E-03 | 2.69E-03 | 2.57E-03 | 2.39E-03 | 2.26E-03 | 2.15E-03 | 1.99E-03 |
| Kidneys | 7.32E-02 | 3.78E-02 | 2.87E-02 | 2.13E-02 | 1.91E-02 | 1.77E-02 | 1.56E-02 |
| Small intestine | 2.14E-02 | 1.64E-02 | 1.55E-02 | 1.44E-02 | 1.38E-02 | 1.32E-02 | 1.28E-02 |
| Left colon | 4.58E-03 | 3.52E-03 | 3.33E-03 | 3.09E-03 | 2.95E-03 | 2.83E-03 | 2.74E-03 |
| Right colon | 9.16E-03 | 7.03E-03 | 6.66E-03 | 6.19E-03 | 5.90E-03 | 5.67E-03 | 5.48E-03 |
| Rectum | 4.58E-03 | 3.52E-03 | 3.33E-03 | 3.09E-03 | 2.95E-03 | 2.83E-03 | 2.74E-03 |
| Prostate (in men) | 9.23E-04 | 4.77E-04 | 3.69E-04 | 3.67E-04 | 3.58E-04 | 3.35E-04 | 2.51E-04 |
| Red marrow | 3.22E-02 | 4.31E-02 | 4.32E-02 | 4.15E-02 | 3.90E-02 | 3.84E-02 | 3.50E-02 |
| Urinary Bladder contents | 1.13E-03 | 5.07E-02 | 6.25E-02 | 7.24E-02 | 6.64E-02 | 6.57E-02 | 6.10E-02 |
| Rest of body | 6.88E-01 | 6.36E-01 | 5.95E-01 | 5.25E-01 | 5.06E-01 | 4.77E-01 | 4.30E-01 |
| Whole body | 9.90E-01 | 9.40E-01 | 8.96E-01 | 8.14E-01 | 7.75E-01 | 7.39E-01 | 6.71E-01 |

**Patient 5:** Normalized activity in source organs for the seven acquired PET scans.

| **Source organ / h after admin** | **0.04** | **0.15** | **0.27** | **0.49** | **0.72** | **0.83** | **1.05** |
| --- | --- | --- | --- | --- | --- | --- | --- |
| Brain | 2.06E-03 | 1.96E-03 | 1.80E-03 | 1.75E-03 | 1.92E-03 | 1.92E-03 | 1.75E-03 |
| Thyroid | 3.00E-04 | 2.46E-04 | 2.09E-04 | 1.76E-04 | 1.45E-04 | 1.42E-04 | 1.20E-04 |
| Lungs | 2.43E-02 | 2.38E-02 | 2.25E-02 | 2.10E-02 | 2.05E-02 | 1.86E-02 | 1.83E-02 |
| Heart contents | 8.45E-03 | 7.16E-03 | 6.61E-03 | 6.05E-03 | 5.14E-03 | 5.06E-03 | 4.78E-03 |
| Liver | 1.28E-01 | 1.31E-01 | 1.28E-01 | 1.22E-01 | 1.09E-01 | 1.04E-01 | 9.42E-02 |
| Stomach contents | 8.11E-03 | 7.90E-03 | 7.21E-03 | 6.61E-03 | 5.46E-03 | 5.13E-03 | 4.25E-03 |
| Spleen | 4.06E-02 | 3.77E-02 | 3.46E-02 | 3.06E-02 | 2.65E-02 | 2.51E-02 | 2.23E-02 |
| Kidneys | 3.03E-02 | 2.65E-02 | 2.30E-02 | 1.89E-02 | 1.59E-02 | 1.42E-02 | 1.26E-02 |
| Small intestine | 3.07E-02 | 2.96E-02 | 2.82E-02 | 2.55E-02 | 2.25E-02 | 2.17E-02 | 1.99E-02 |
| Left colon | 6.58E-03 | 6.34E-03 | 6.04E-03 | 5.47E-03 | 4.83E-03 | 4.65E-03 | 4.26E-03 |
| Right colon | 1.32E-02 | 1.27E-02 | 1.21E-02 | 1.09E-02 | 9.66E-03 | 9.30E-03 | 8.52E-03 |
| Rectum | 6.58E-03 | 6.34E-03 | 6.04E-03 | 5.47E-03 | 4.83E-03 | 4.65E-03 | 4.26E-03 |
| Prostate (in men) | - | - | - | - | - | - | - |
| Red marrow | 1.09E-02 | 1.15E-02 | 1.00E-02 | 9.87E-03 | 9.31E-03 | 7.83E-03 | 7.10E-03 |
| Urinary Bladder contents | 0.00E+00 | 0.00E+00 | 4.65E-05 | 1.16E-03 | 6.18E-03 | 6.32E-03 | 6.90E-03 |
| Rest of body | 6.75E-01 | 6.41E-01 | 6.15E-01 | 5.63E-01 | 5.20E-01 | 5.02E-01 | 4.61E-01 |
| Whole body | 9.86E-01 | 9.45E-01 | 9.02E-01 | 8.29E-01 | 7.63E-01 | 7.31E-01 | 6.71E-01 |

**Patient 6:** Normalized activity in source organs for the seven acquired PET scans.
