# Supplementary material for: Radiation dosimetry of 18F-AzaFol: A first in-human use of a folate receptor PET tracer
Source: EJNMMI Res. 2020 Apr 8;10:32. doi: 10.1186/s13550-020-00624-2 (PMC7142191; doi:10.1186/s13550-020-00624-2)

**Figure S6.** Time-activity curves (TAC), %IA/g and %IA/g corrected for ^18^F physical decay for all tumor analyzed (T#). In bracket, we indicated the patient identifier (P#).


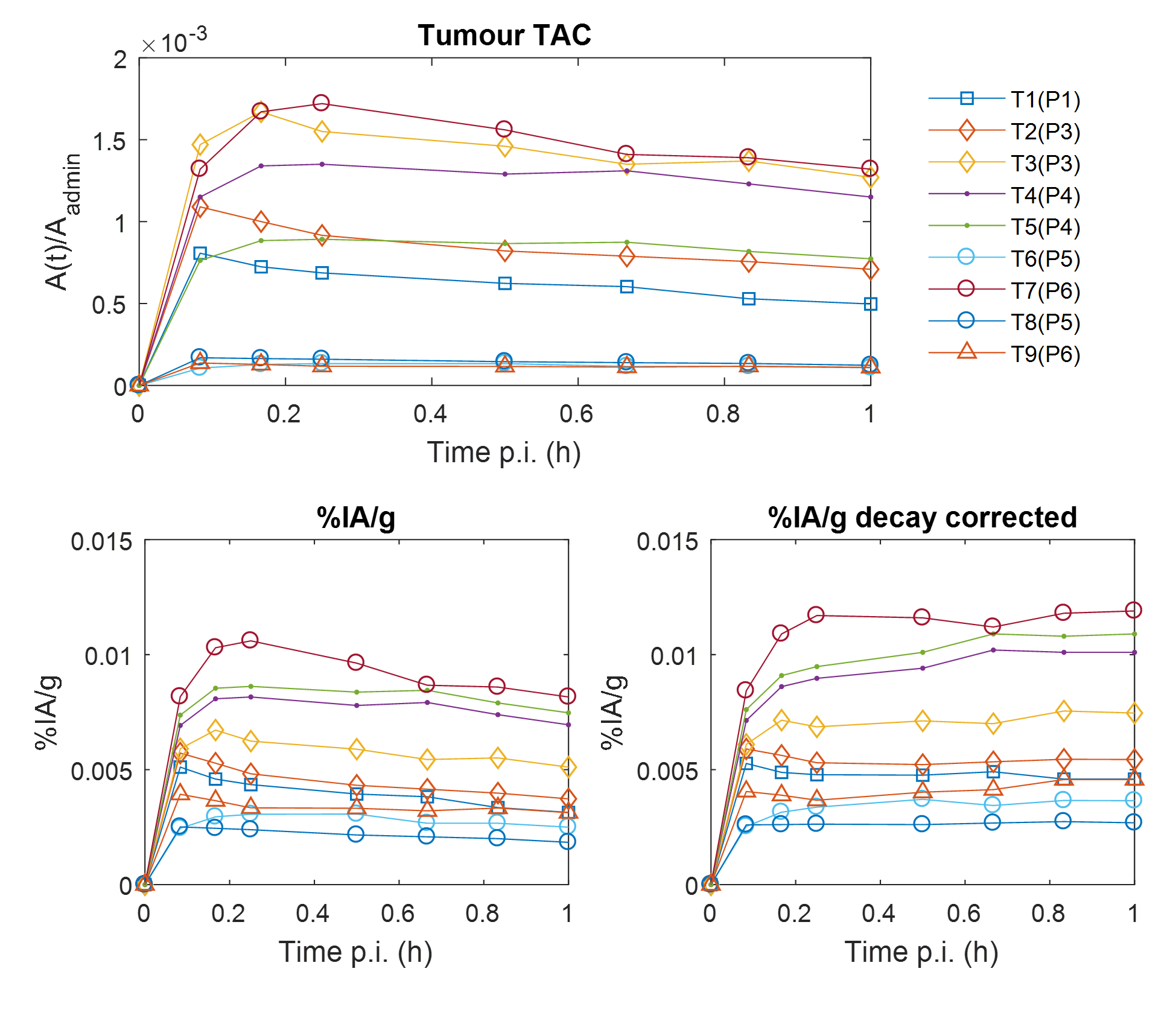

Supplement: Supplementary file 6 — Additional file 6: Figure S6. Time-activity curves (TAC), %IA/g and %IA/g corrected for 18F physical decay for all tumor analyzed (T#). In bracket, we indicated the patient identifier (P#). [file 13550_2020_624_MOESM6_ESM.docx]
